# Goal-Dependent Use of Temporal Regularities to Orient Attention under Spatial and Action Uncertainty

**DOI:** 10.5334/joc.360

**Published:** 2024-04-25

**Authors:** Irene Echeverria-Altuna, Anna C. Nobre, Sage E. P. Boettcher

**Affiliations:** 1 Department of Experimental Psychology, University of Oxford, Oxford, United Kingdom; 2 Oxford Centre for Human Brain Activity, Wellcome Centre for Integrative Neuroimaging, Department of Psychiatry, University of Oxford, Oxford, United Kingdom; 3 Department of Psychology, Yale University, United States of America

**Keywords:** Attention, Statistical Learning, Temporal Expectations, Temporal Attention, Spatial Attention, Motor Preparation, Vision

## Abstract

The temporal regularities in our environments support the proactive dynamic anticipation of relevant events. In visual attention, one important outstanding question is whether temporal predictions must be linked to predictions about spatial locations or motor plans to facilitate behaviour. To test this, we developed a task for manipulating temporal expectations and task relevance of visual stimuli appearing within rapidly presented streams, while stimulus location and responding hand remained uncertain. Differently coloured stimuli appeared in one of two concurrent (left and right) streams with distinct temporal probability structures. Targets were defined by colour on a trial-by-trial basis and appeared equiprobably in either stream, requiring a localisation response. Across two experiments, participants were faster and more accurate at detecting temporally predictable targets compared to temporally unpredictable targets. We conclude that temporal expectations learned incidentally from temporal regularities can be called upon flexibly in a goal-driven manner to guide behaviour. Moreover, we show that visual temporal attention can facilitate performance in the absence of concomitant spatial or motor expectations in dynamically unfolding contexts.

## Introduction

Consistent temporal structuring of events is prevalent in daily experience, ranging from regular isochronous rhythms (e.g., footsteps) to more complex sequences (e.g., music). These temporal regularities embedded in our environments give rise to expectations about *when* events are likely to occur, which, in turn, proactively guide selective anticipatory attention (for reviews see Correa, 2010; Nobre & Rohenkohl, 2014; Nobre & van Ede, 2018, 2023; Vangkilde et al., 2013).

Selective attention comprises the various neural and psychological functions that selectively prioritise information for processing to guide adaptive behaviour through anticipation, selection, or gating of signals according to task goals, expectations, sensory salience, or various forms of memory (see Nobre, 2018). Visual temporal attention refers to the prioritisation of visual information at relevant moments in time, facilitating perception and/or action in our dynamic world. In the present study, we consider how two fundamental factors guide the prioritisation of stimuli – expectations and task goals.

Visual temporal expectation has been suggested to exert its effects by latching onto other relevant anticipated stimulus attributes and modulating them at predictable times (Nobre & Rohenkohl, 2014). Joint expectations about the location and timing of a stimulus (spatiotemporal expectations) have consistently been reported to facilitate performance (e.g., Boettcher et al., 2022; Doherty et al., 2005; MacKay & Juola, 2007; Rieth & Huber, 2013; Rolke et al., 2016; Seibold et al., 2020; Weinbach et al., 2015). Furthermore, temporal expectation modulates the firing rates of neurons coding the locations of anticipated task-relevant stimuli (e.g., Ghose & Maunsell, 2002; Janssen & Shadlen, 2005). At the extreme, some studies have reported that visual temporal expectation in the absence of spatial certainty fails to facilitate behaviour, instead acting only by amplifying the effects of spatial expectations (e.g., O’Reilly et al., 2008; Rohenkohl et al., 2014).

In parallel, temporal expectation has been proposed to act by modulating preparatory motor activity at specific times, thus speeding response times (Boettcher et al., 2021; Heideman, et al., 2018; Miniussi et al., 1999; Thomaschke & Dreisbach, 2013; Trillenberg et al., 2000; van Ede et al., 2020; van Elswijk et al., 2007). However, it is debated whether temporal expectation modulates specific motor programs, or instead acts by exerting a more general influence on motor processes (Cotti et al., 2011; Kornysheva et al., 2013; Shin & Ivry, 2002). Indeed, in most studies investigating visual temporal expectation, the locations of expected targets and/or the required responses are kept constant or predictable. Consequently, it is currently unknown whether allocating attention to points in time based on temporal expectation can facilitate behaviour in the absence of spatial or action-related certainty.

The effects of visual temporal attention on behaviour have been studied using different experimental approaches. Tasks using predictive or instructive temporal cues (Coull & Nobre, 1998; Denison et al., 2017; Griffin et al., 2002; Nobre, 2001) have grounded most of the research on visual temporal attention. Like their spatial counterpart, temporal cueing tasks use cues to indicate when a stimulus will likely be displayed (e.g., Coull & Nobre, 1998) or which stimulus is goal-relevant based on its timing (Denison et al., 2017, 2021; Fernández et al., 2019; Griffin et al., 2002). In turn, the predictive or instructive cues deploy endogenous attention to moments in time (for reviews see Correa, 2010; Nobre, 2010). In contrast, in most everyday situations, attention is typically guided by predictions formed through the incidental learning of regularities in the environment. In the case of spatial attention, repeated contextual spatial configurations can guide attention to specific locations (Chun & Jiang, 1998). Similarly, targets preceded by repeating temporal patterns are more readily attended and better detected (Olson & Chun, 2001).

Task designs using informative endogenous cues vs. incidental contextual learning capture complementary aspects of how temporal expectations are formed and utilised in real-world behaviour (Tal-Perry & Yuval-Greenberg, 2022). On the one hand, contextual cueing tasks simulate real-world scenarios by emphasizing the incidental learning of temporal regularities that can guide expectation-based attention (Beck et al., 2014; Boettcher et al., 2022; Heideman et al., 2018; O’Reilly et al., 2008; Rieth & Huber, 2013; Salet et al., 2021). In contrast, informative cueing tasks allow for trial-wise manipulations of attention based on task goals, hence probing the flexible and goal-dependent usage of temporal expectations (Denison et al., 2017, 2021; Fernández et al., 2019; Griffin et al., 2002). Both the automatic utilisation of incidentally learned temporal regularities and flexible, goal-dependent prioritisation are likely to be essential features of how temporal expectations guide behaviour in our dynamically unfolding environments (Nobre & van Ede, 2018).

In the present study, we built on the strengths of both approaches by developing a design that encourages goal-dependent usage of contextually embedded temporal expectations to identify targets within extended dynamic backgrounds with pronounced sensory competition. In the task, participants formed temporal expectations based on the characteristic temporal regularities of differently coloured stimuli appearing within two rapidly presented concurrent streams. The identity of the relevant target (based on colour) on any given trial was cued on a trial-by-trial basis to test whether participants could utilise learned temporal regularities flexibly to prioritise the task-relevant stimulus. Importantly, the exact stimulus location (left or right stream) and required response hand were unpredictable and varied trial-by-trial. Across two experiments, we investigated whether temporal expectation, independent of spatial and motor expectations, facilitated behaviour. To foreshadow our results, we found that visual temporal expectation can be flexibly directed to task-relevant events in the absence of spatial or response-related certainty.

## Experiment 1: online study

### Methods

#### Participants

This study was approved by the Central University Research Ethics Committee of the University of Oxford (R73580/RE001). We collected data from 54 participants recruited through the Prolific Academic platform (https://www.prolific.co). Pre-screening of participants ensured they were aged 18 to 40, fluent in English, had normal or corrected eyesight, and fulfilled specific participation requirements in Prolific Academic. Study inclusion required an approval rate above 95% and participation in at least ten prior experiments. Participants were paid at a rate of £7.50 per hour and received a bonus of up to £2.50, which scaled with performance above 90% accuracy. On average participants received a bonus payment of £1.50. All participants provided informed consent prior to beginning the task. To accommodate the additional variability in online testing, we chose our sample size by doubling the size of the samples used in similar in-person studies of the effects of temporal expectations in extended contexts (i.e., Boettcher et al., 2022).

Data quality in online testing can vary (Sauter et al., 2020), prompting the adoption of strict exclusion criteria. Participants were excluded from further analysis if their performance accuracy was below 60%, did not respond to more than 30% of trials, or had mean RTs above 5 standard deviations from the across-participant mean. We excluded 5 participants in total, leaving 49 for analyses (14 female; mean age: 24.75, SD: 7; 8 left-handed).

#### Task and procedure

##### Online experiment

The experimental script was generated using PsychoPy (PsychoPy Builder v2021.1.4; Peirce et al., 2019) and hosted online through Pavlovia (https://pavlovia.org/; Sauter et al., 2020). Participants completed the study on their personal computers or laptops. Participants were encouraged to use Mozilla Firefox or Google Chrome for this study and asked to keep 60 cm away from their screens. They were not allowed to take part in the study from their phones or tablets. At the beginning of the experiment, participants scaled the image of a credit card to match the size of a physical one which they placed against the screen. This procedure was used to estimate the resolution of their computer screen before starting the experiment (Li et al., 2020). The ratio between the card image width (pixels) and the actual card width (cm) estimated the pixel density (pixel per cm) per participant. Together, this value and the recommended distance from the screen (60 cm) allowed us to estimate the degrees of visual angle of stimulus presentation.

##### Stimuli and experimental procedure

**Figure 1:**
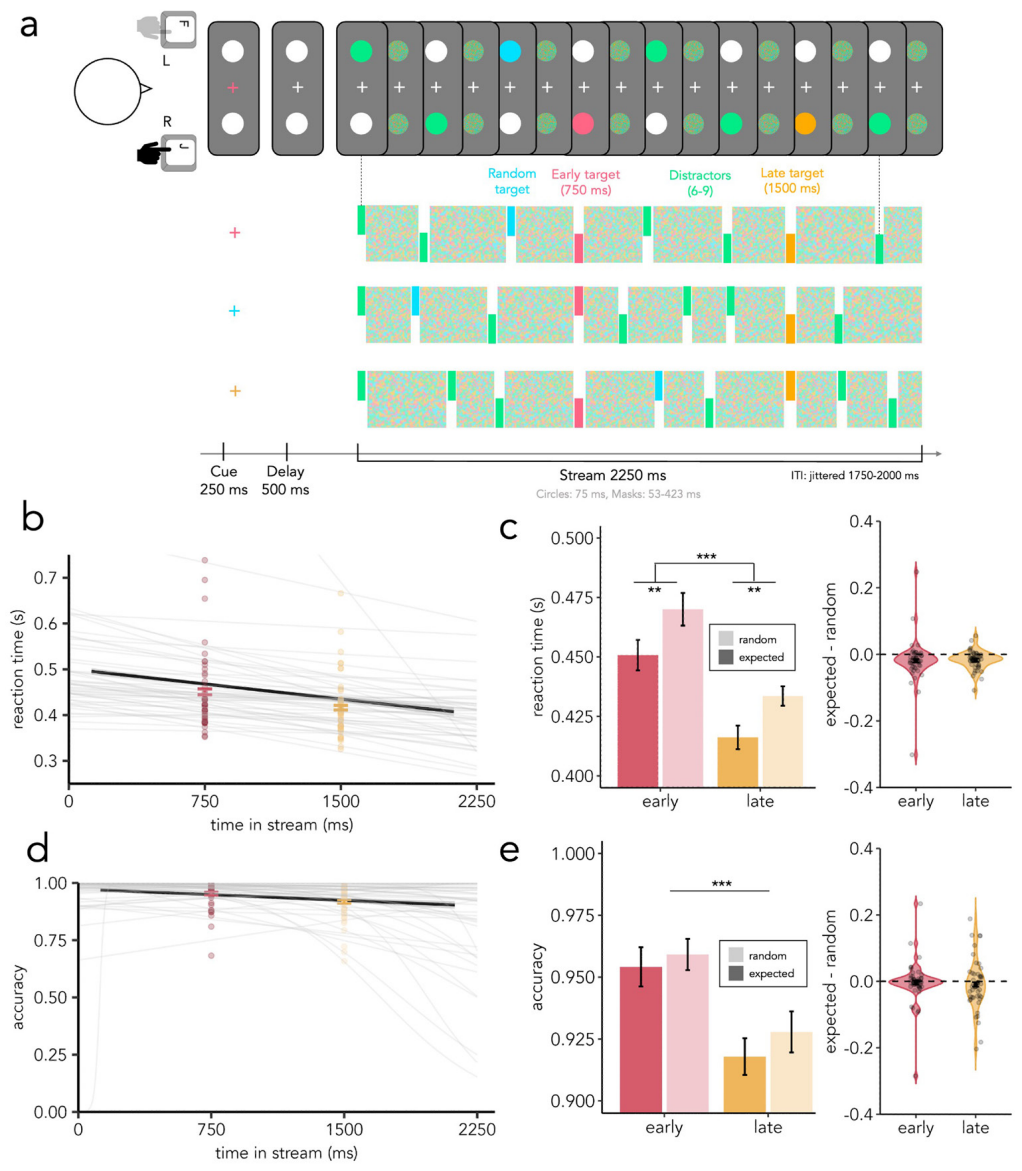
**Online task design, accuracy, and RT results. a)** Design of the online experiment. At the beginning of each trial, participants saw a cue (change in the colour of the fixation cross) indicating which of three coloured targets to detect. Participants had to search for a circle with this colour within two streams of successively appearing circles and masks. Unknown to the participants, one of these targets appeared at 750 ms from stream onset (early target), another appeared at 1500 ms from stream onset (late target), and the other could happen at any time (random target). Participants had to respond with the hand corresponding to the side of the trial-designated target. **b)** Mean RT to random targets as a function of target onset as estimated by participant-specific GLMs (thin grey lines) and actual RT to early (pink) and late (yellow) targets across participants (error bars represent standard error of the mean (SEM) and points represent individual participants). **c)** Left: average RT to expected targets (actual) and average RT to random targets (as estimated by participant-specific GLMs). Error bars represent SEM, pink represents early time, yellow represents late time, lighter colours represent estimated (random) RTs and darker colours represent actual (expected) RTs. Statistical significance is indicated with asterisks. Right: difference between RT at the time of early/late target as estimated from participant-specific GLMs and actual RT to early/late targets. Individual participants’ differences are depicted by dots, error bars represent the SEM and dashed lines represent 0 (no difference). **d)** Mean accuracy to random targets as a function of target onset as estimated by participant-specific GLMs (thin grey lines) and actual accuracy to early (pink) and late (yellow) targets across participants (error bars represent standard error of the mean (SEM) and points represent individual participants). **e)** Left: average accuracy to expected targets (actual) and average accuracy to random targets (as estimated by participant-specific GLMs). Error bars represent SEM, pink represents early time, yellow represents late time, lighter colours represent estimated (random) accuracies and darker colours represent actual (expected) accuracies. Statistical significance is indicated with asterisks. Right: difference between accuracy at the time of early/late target as estimated from participant-specific GLMs and actual accuracy to early/late targets. Individual participants’ differences are depicted by dots, error bars represent the SEM and dashed lines represent 0 (no difference).

The task involved identifying one of up to three possible coloured targets that could appear within either of two concurrent rapidly presented streams. Possible targets were coloured circles (green, blue, pink, or yellow), which appeared only once, within either stream, during the trial. Which colour was relevant for the trial was cued by a coloured central cue at trial onset, and participants produced a speeded localisation response by using the equivalent (left vs. right) hand upon detecting the relevant target (Figure 1a). Importantly, the location of target appearance (left vs. right) was equiprobable and, consequently, the required response hand was also unpredictable. The task was divided into six blocks, each containing 72 trials and lasting ~6 min. Before beginning the first block, participants conducted a practice block of 24 trials. Participants were instructed to rest between blocks. In total, the task lasted around 1 hr.

At the beginning of each trial, a centrally presented cue indicated which of the possible target colours required a response on that trial. The cue was a 250-ms change in the colour of the central fixation cross to match the colour of the designated target. After a 500-ms delay, participants were presented with two streams of coloured and white circles (diameter: 5 degrees of visual angle; DVA). Coloured circles appeared briefly (75 ms) either on the left or on the right side of the screen with a white circle displayed on the opposite side at an estimated distance of 8 DVA from the centre of the screen. Both circles were immediately followed by a bilateral multi-coloured mask, which remained on the screen until the onset of the next coloured circle (Figure 1).

Each circle within the streams was coloured with one out of four possible colours, which were equidistantly spaced in the CIELab colour space (CIE, 2004; green: #01EB87, orange: #FFAC00, blue: #00E1FF, red: #FF6686). One of these colours was designated as the *distractor* colour, and the other three corresponded to the three possible *target* colours. Designated target and distractor colours were constant within a participant and were counterbalanced across participants. On each trial, participants were cued to identify one out of the three target colours. Thus, only the cued target was task-relevant in each trial, but uncued, task-irrelevant, targets and distractors were also displayed within the streams.

As the main experimental manipulation of temporal expectation, the three targets differed according to *when* they appeared. The temporal predictability of targets was linked to their colour. *Early* targets appeared at a fixed time of 750 ms from stream onset; *late* targets appeared at a fixed time of 1500 ms from stream onset; and *random* targets could appear at any time from stream onset, except for around the *early* (700–800 ms) and *late* (1450–1550 ms) time points.

Participants responded indicating the location of the circle that matched the cue colour shown at the start of each trial. All circles had an equal probability of appearing on the left or on the right and there was a balanced number of left and right circles in each trial +/– 1. As soon as they detected the target, participants had to press either the left (*f)* or the right (*j)* key corresponding to the location of the target in the left or right stream, respectively. Importantly, because of the dependency between target location and response hand, the required response was random and unpredictable until the target appeared. For target colours with consistent (early or late) temporal onsets, we hypothesised that participants would incidentally learn the time of target appearance and proactively direct attention to the predicted moment of the target colour to identify target location (left vs. right) and generate the correct response (left vs. right hand). In contrast, the lack of temporal prediction associated with the randomly timed targets would not support temporally focused proactive attention, resulting in a relative behavioural disadvantage in the detection of randomly timed targets compared to temporally predicted targets.

The streams had a total duration of 2250 ms and did not stop when participants responded. During inter-trial intervals (ITIs; jittered 1750–2000 ms), two placeholder white circles were displayed on the left and the right of the screen. Participants were instructed to keep their eyes on the central fixation cross for the duration of each block.

To minimise the potential effects of foreperiod-linked expectations to detect the random targets, their onset probability approximated a non-ageing flat distribution (Trillenberg et al., 2000). The absolute likelihood for the random target to appear was 50% during the first third of the trial, 25% in the middle third, and 12.5% in the final third **(Supplementary Figure 1)**. Thus, as time passed, the conditional probability (hazard rate) for the target to occur in each third of the trial, had it not yet appeared, remained at 50%.

In each trial, participants had to respond to the target with the cued colour. Participants were cued to respond to the early, late, and random targets equiprobably (33.3% each; **Supplementary Figure 1**). In 37.5% of trials, one of the three targets was absent (12.5% early absent, 12.5% late absent, 12.5% random absent). These trials did not overlap. A third of the trials in each absent category occurred during a trial with a corresponding cue. This resulted in 4.17% of early/late/random trials in which participants were cued on the corresponding target and the target was absent. In these trials, participants were instructed not to respond. Overall, a total of 12.5% of all trials required no response from participants **(Supplementary Figure 1)**. No-response trials were included to promote participants’ engagement in this task and to preserve the non-ageing flat distribution of random target appearance.

All trials contained between 9–12 coloured circles. Most trials contained 3 targets and 6–9 distractors. However, when one of the targets was absent, the trial instead contained 2 targets and 7–10 distractors. The number of distractors was selected randomly from a uniform distribution. To distribute the stimuli equally across the duration of the trial, the streams were segmented into three-thirds. The early target marked the onset of the middle third and the late target marked the onset of the final third. Either 3 or 4 coloured circles appeared in each third. The first circle appearing in each trial was always a distractor and different numbers of stimuli could precede each target, hence minimising any effects of temporal order and emphasizing temporal regularities based on onset timings instead.

Each coloured circle lasted 75 ms. During the inter-stimulus intervals (ISIs), bilateral circular masks were displayed. ISIs were calculated by choosing pseudo-random numbers from a gamma distribution with a minimum duration of 53 ms, a maximum of 423 ms, and an average of 141 ms, depending on how many stimuli were presented in each third. Twelve unique masks were created by sampling 64 × 64 elements from a uniform distribution of all target and distractor colours, and one of these masks (chosen randomly) appeared in each trial.

At the beginning of the task, participants were shown the three target colours they would need to detect throughout the study. At the end of the session, they were asked to fill in a questionnaire prompting them to report any patterns they had noticed regarding the appearance of the stimuli. Participants had unlimited time to respond using free text. Additionally, participants were shown a horizontal line representing “time in trial” and were asked to place a rectangle at the location on the line that corresponded to the estimated time of target appearance. The horizontal line was divided into 10 bins and participants were asked to report the time of each target’s appearance on a different line. We calculated the percentage of participants who placed the rectangle on bins 3 or 4 out of 10 when asked about the timing of the early target. Similarly, we calculated the percentage of participants who placed the rectangle on bins 6 or 7 out of 10 when prompted to report the time of late target occurrence. This approach yielded a chance-level report of target timing of 20%.

#### Data analysis

##### Data cleaning

All data analyses were performed using R studio (RStudio Team, 2020). All analyses were performed on trials in which the target stimulus was present. The following trials were excluded from reaction time (RT) analyses: incorrect trials, trials with responses faster than 100 ms, and trials with responses slower than three times the standard deviation (SD) of the average RT per participant and condition (early, late, and random) following removal of incorrect trials and trials with RTs faster than 100 ms. After cleaning, an average of 3.43% (SD: 3.54%) trials were removed per participant from the RT analyses.

##### Variables of interest

Two dependent variables were of interest: RT (time from target onset to correct response) and accuracy (proportion of correct responses out of all responses). All analyses compared these measures according to the three conditions: early, late, and random targets. The number of trials in which participants responded when the cued target was absent (i.e., false alarms) was very low (an average of 0.037% of trials; range: 0–0.12%). Consequently, false alarms were not further analysed in the present study.

In a supplementary analysis, we combined reaction time and accuracy into the inverse efficiency score metric (IES; Townsend & Ashby, 1983) to assess the potential effects of a speed-accuracy trade-off on participants’ performance over time. The IES is defined as the average reaction time in correct trials divided by the proportion of correct responses per trial and per condition. We calculated the IES for early and late targets per participant and assessed the effect of early/late on this metric with a paired-sample t-test. To calculate the IES to random targets, we divided the time from stream onset until stream offset into 6 time-bins ensuring the same number of random targets per bin and assessed the effects of time-in-stream on IES with an ANOVA (see **Supplementary Figure 4**).

Importantly, previous studies have shown that RT and accuracy change as a function of time-in-trial before target appearance, the foreperiod, with RT typically decreasing as a function of time (Los, 2010; Luce, 1991; Niemi & Naatanen, 1981). Given that the main distinguishing feature among the three conditions in the task was the time of target appearance (early, late, and random), we addressed potential confounds arising from foreperiod effects in our analyses. This was achieved by incorporating the effects of the onset times for the random target on performance in two different ways.

##### Participant-specific Generalised Linear Modelling, followed by parametric comparisons

For each participant, we fit a generalised linear model (GLM; using the *glm* function from *lme4* R package; Version 1.1–30; Bates et al., 2015) to the RTs in response to random targets (assuming a Gamma distribution of RTs and a Gaussian link function; Lo & Andrews, 2015) and to the accuracy of the responses to random targets (assuming a binomial distribution of accuracy and a Gaussian link function). We modelled RT and correctness for random targets as a linear function of random-target onset time. Additional analyses also considered other, non-linear link functions (see **Supplementary Figures 2** and **3**). In participant-specific accuracy GLMs, a Gaussian link function was used when the model converged and, alternatively, a logit link function was chosen in 27 of the models.

From each participant-specific model of performance to random targets, we interpolated the value of RT and a value of accuracy at the time of early (750 ms from stream onset) and late (1500 ms from stream onset) target appearance. Subsequently, we compared the interpolated or estimated RT and accuracy values when participants responded to random targets with the actual RT and accuracy values when participants responded to early or late targets. We hypothesised that participants would be faster and more accurate in responding to temporally predictable targets than what would have been estimated based on foreperiod effects alone. To test this hypothesis, we assessed how the factors of predictability (predicted versus random) and time (early versus late) modulated RT and accuracy (actual and estimated) using 2 × 2 analyses of variance (ANOVAs) (Figure 1).

To gauge the consistency of the results in our participant-specific GLMs, we repeated the same procedure using different link functions in supplementary analyses. We tested the relation between RT and random-target onset assuming linear, logarithmic and inverse link functions for all participants and by choosing the best-fitted model (out of the ones above) for each participant **(Supplementary Figure 2b, c)**. Similarly, we modelled participants’ accuracy as a function of random-target onset with four different link functions: linear, logit, cauchit, and the participant-specific best fit **(Supplementary Figure 2d, e)**. The best-fitted model was identified by selecting the model with the minimal Bayesian information criterion (BIC) and Akaike information criterion (AIC) values.

##### Generalised Linear Mixed-Effects Models

To confirm the reliability of our results, we built two comprehensive generalised linear mixed-effects models (GLMMs) for RTs and accuracies, respectively. These included condition and target onset as predictors, together with the random effects of participant and target colour.

We modelled RT according to the following formula as implemented in *lmer4*, assuming a gamma distribution of RT and an identity link function between RT and the fixed effects.


\[
\begin{array}{c}
RT\sim 1{ + }\;Condition\;*\;TargetOnset\\
{ + }\;\left( {1\;{ + }\;Condition\;*\;TargetOnset\;{|}\;Participant} \right)\\
{ + }\;\left( {1\;{ + }\;Condition\;{*}\;TargetOnset\;|\;TargetColour} \right)
\end{array}
\]

The *Condition* variable had three levels according to the timing of target onset in each trial: early, late, or random. We used the random condition as the reference contrast, ensuring comparison of the early and late conditions to the random condition. Furthermore, adding the *TargetOnset* variable as a predictor captured any variability related to foreperiod effects that might affect the detection of the random target. Consequently, any significant effect of condition on RT would represent effects of visual temporal expectation, above and beyond foreperiod effects and random effects arising from participant and target colour.

Following Matuschek et al.’s (2017) procedure, and with the aim of balancing Type-I errors and power, we simplified this maximal model by eliminating predictors that explained little to no variance until the most parsimonious model remained that did not significantly worsen the model fit:


\[
\begin{array}{c}
RT\sim 1{ + }\;Condition\;*\;TargetOnset\\
{ + }\left( {1\;{ + }\;Condition\;*\;TargetOnset\;|\;Participant} \right)
\end{array}
\]

We followed a similar procedure for accuracy, assuming a binomial distribution of the dependent variable and an identity link function between correctness and the fixed effects:


\[
\begin{array}{c}
Correctness\sim 1{ + }\;Condition\;*\;TargetOnset\\
{ + }\;\left( {1\;{ + }\;Condition\;*\;TargetOnset\;|\;Participant} \right)\\
{ + }\;\left( {1\;{ + }\;Condition\;*\;TargetOnset\;|\;TargetColour} \right)
\end{array}
\]

Like above, we simplified this maximal model by eliminating predictors until we were left with the most parsimonious model that fit our data best:


\[
\begin{array}{c}
Correctness\sim 1{ + }\;Condition\;*\;TargetOnset\\
{ + }\left( {1\;{ + }\;Condition\;*\;TargetOnset\;|\;Participant} \right)
\end{array}
\]

All models were estimated using a maximum likelihood criterion. We report the outputs of the models as unstandardised regression coefficients with the t-statistics and the results of two-tailed tests with a 5% criterion for significance.

Due to participant-specific differences in foreperiod effects (e.g., changes in RT/accuracy variance over time) we decided to base our main interpretations on the participant-specific GLMs and considered the results of the GLMMs as complementary and confirmatory.

### Results

The main experimental question concerned whether participants were faster and more accurate at detecting temporally expected targets compared to temporally unexpected targets. Such an effect would indicate benefits of visual temporal expectation in the absence of certainty about stimulus location (left or right stream) or required response (left or right hand). To address this question, RTs and accuracies for the early and late expected targets were compared to the estimated RT and accuracy values at early and late times based on random-target onset (see *Methods*).

A 2 × 2 ANOVA on response times revealed a main effect of time in trial (F(1,48) = 27.25, ***p < .001, η^2^ = .04) and a main effect of predictability (F(1,48) = 9.28, **p = .003, η^2^ = .01) on RT, but no interaction between the factors (F(1,48) = .06, p = .8, η^2^ < .000). The main effect of time is suggestive of foreperiod effects in this task, with participants responding faster to late targets than early targets. Additionally, the main effect of predictability indicates that participants were faster at detecting temporally expected targets than temporally unexpected ones.

For accuracy, we found only a main effect of time (F(1,48) = 15.1, ***p < .001, η^2^ = .06), with no main effect of predictability (F(1,48) = 0.78, p = .38, η^2^ = .003) or interaction between the factors (F(1,48) = .27, p = .6, η^2^ = .0003) (Figure 1e). Accuracy deteriorated with time in the trial. We speculated that the absence of effects of temporal predictability on accuracy in the present task may have resulted from the near-ceiling performance of participants, especially, towards the beginning of the streams.

We observed a similar pattern of results when fitting the participant-specific GLMs assuming a set of non-linear relations between the dependent variables (RT and accuracy) and target onset **(see Supplementary Figure 2)**.

To test the presence of systematic changes in performance as a function of time in trial, we combined RT and accuracy into a single measure of inverse efficiency score (IES; Townsend & Ashby, 1983). A paired-sample t-test revealed no differences in IES between early and late targets (t(48) = 1.47, p = 0.15, d = .21; **Supplementary Figure 4**). We additionally divided random targets into 6 time-bins depending on their onset with respect to stream onset and calculated IES per bin and per participant. An ANOVA with bins as a factor revealed a small, yet significant effect of bin on IES (F(1,48) = 4.36, *p = .04, η^2^ = .083) and a follow-up linear contrast revealed a downwards slope of IES as a function of binned time (β = –.008, t = –2.16, *p = .03). From this, we concluded that time-in-stream changed response properties (i.e., speed-accuracy trade-off) and had a slight effect on overall task performance in Experiment 1 **(Supplementary Figure 4)**.

Similar results were obtained when fitting the dependent variables in two exhaustive GLMMs that considered RT (*early*: β = –.022, t = –1.56, p = .12; *late*: β = –.019, t = –3.22, **p = .001) and accuracy (*early*: β = .023, z = .26, p = .79; *late*: β = –.07, z = –.84, p = .4) as the dependent variables and participants as a random effect (see *Methods* for details). The small disparities observed in the results revealed by each statistical approach likely arise from differences in how participant-specific effects are integrated and considered in each kind of model. While GLMMs are a powerful method for integrating single-trial variables into a model, they may not explicitly consider participant-specific differences in performance (e.g., such as participant-specific changes in RT/accuracy variance as a function of time), which may contribute to the present differences. Due to their sensitivity to participant-specific performance patterns, our conclusions are based on the results of the participant-specific GLMs while the GLMMs serve to provide supplementary confirmation.

In the post-experiment questionnaire, three participants out of forty-nine reported having noticed that some target colours appeared closer to the onset and others closer to the offset of the streams. When prompted to place a vertical bar on a horizontal line depicting “time in trial”, 18% of the participants placed the early target colour around the time of its appearance and 13% of the participants placed the late colour correctly. Chance level placement would occur on 20%. This suggests that, for most participants, temporal regularities within the trial were learned incidentally and benefited performance implicitly, without their explicit knowledge.

## Experiment 2: in-person study

### Methods

The second experiment was aimed at replicating the results from Experiment 1 during in-person testing and after introducing minor modifications to improve task sensitivity and offer generalization. Specifically, we increased the difficulty of the task to enhance the variability of participants’ accuracies and thereby optimise the design to investigate any potential effects on target detection accuracy. Additionally, to ensure that the results found in Experiment 1 were not tied to the specific choices of colours and timings, we chose four different colours and slightly different stimulus durations and stream timings in Experiment 2.

#### Participants

This study was approved by the Central University Research Ethics Committee of the University of Oxford (R76234/RE001). Due to reports of varying data quality in online experiments (Sauter et al., 2020), we reduced the sample size from Experiment 1 to Experiment 2. We chose our sample size based on the effect sizes reported in similar studies studying the effects of temporal expectations in dynamic and extended contexts (i.e., Boettcher et al., 2022). We collected data from 24 participants recruited through the Oxford Psychology Research (OPR) participant recruitment scheme. Pre-screening of participants ensured they were aged 18 to 40, fluent in English, had normal or corrected eyesight and hearing, were not taking psychoactive medication, and had no history of severe neurological or psychiatric disorders. Participants were paid at a rate of 10 £ per hour. All participants provided informed consent prior to beginning the study. Using the same participant exclusion criteria as above, we excluded one participant from further analyses, leaving 23 for analyses (11 female; mean age: 25.96, SD: 3.4; 5 left-handed).

#### Task and procedure

##### In-person experiment

The experimental script was generated using PsychoPy (PsychoPy Builder v2021.1.4; Peirce et al., 2019). To perform the task, participants were placed 60 cm away from a monitor [22-inch (55.88 cm) Samsung SyncMaster 2233; resolution, 1680 × 1050 pixels; refresh rate, 100 Hz; screen width, 47 cm].

##### Stimuli and procedure

**Figure 2:**
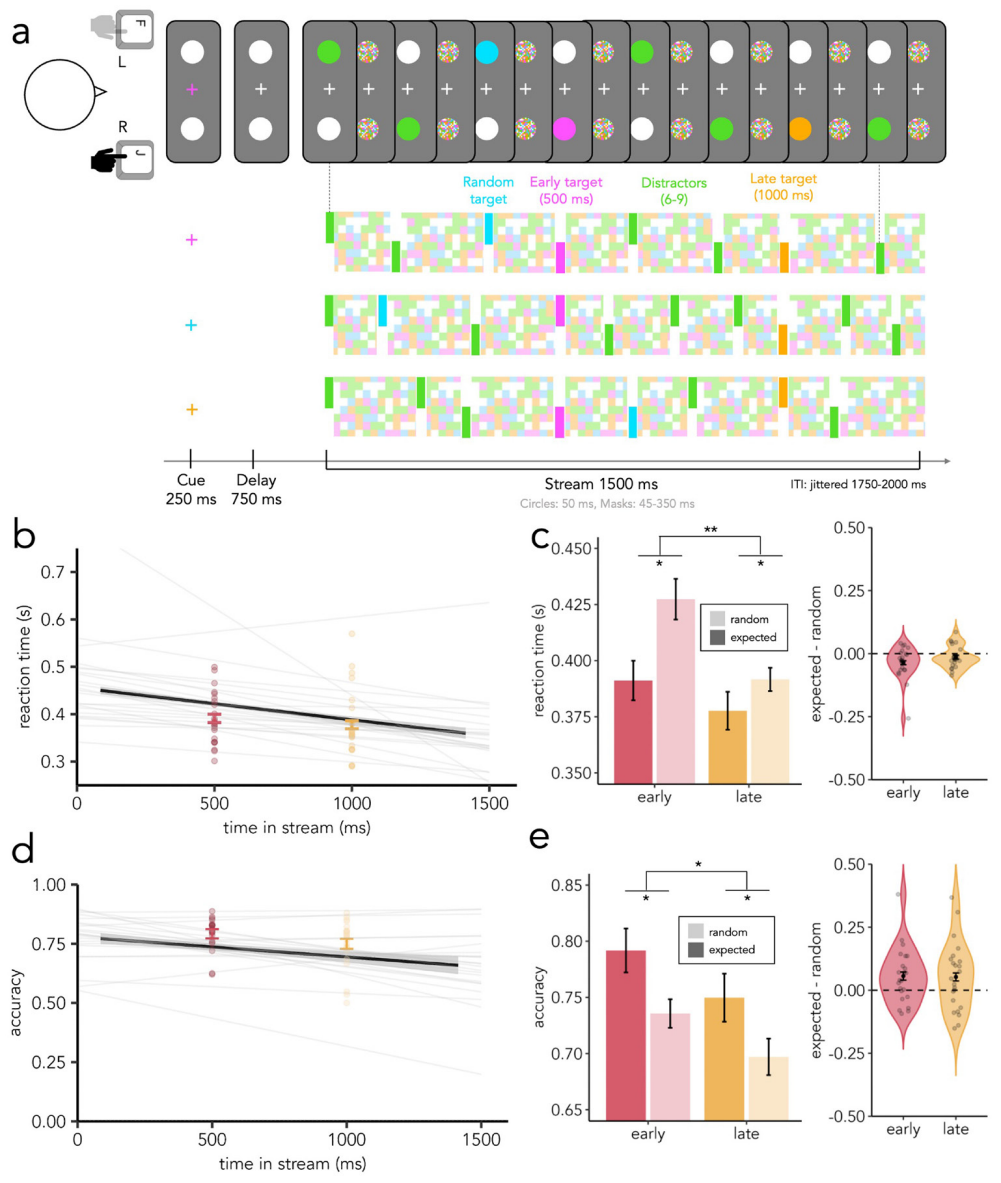
**In-person task design, accuracy, and RT results. a)** Design of the in-person experiment. At the beginning of each trial, participants saw a cue (change in the colour of the fixation cross) indicating which of three coloured targets to detect. Participants had to search for a circle with this colour within two streams of successively appearing circles and masks. Unknown to the participants, one of the coloured targets appeared at 500 ms from stream onset (early target), another appeared at 1000 ms from stream onset (late target), and the other could happen at any time (random target). Participants had to respond with the hand corresponding to the side of the trial-designated target. **b)** Mean RT to random targets as a function of target onset as estimated by participant-specific GLMs (thin grey lines) and actual RT to early (pink) and late (yellow) targets across participants (error bars represent standard error of the mean (SEM) and points represent individual participants). **c)** Left: average RT to expected targets (actual) and average RT to random targets (as estimated by participant-specific GLMs). Error bars represent SEM, pink represents early time, yellow represents late time, lighter colours represent estimated (random) RTs and darker colours represent actual (expected) RTs. Statistical significance is indicated with asterisks. Right: difference between RT at the time of early/late target as estimated from participant-specific GLMs and actual RT to early/late targets. Individual participants’ differences are depicted by dots, error bars represent the SEM and dashed lines represent 0 (no difference). **d)** Mean accuracy to random targets as a function of target onset as estimated by participant-specific GLMs (thin grey lines) and actual accuracy to early (pink) and late (yellow) targets across participants (error bars represent standard error of the mean (SEM) and points represent individual participants). **e)** Left: average accuracy to expected targets (actual) and average accuracy to random targets (as estimated by participant-specific GLMs). Error bars represent SEM, pink represents early time, yellow represents late time, lighter colours represent estimated (random) accuracies and darker colours represent actual (expected) accuracies. Statistical significance is indicated with asterisks. Right: difference between accuracy at the time of early/late target as estimated from participant-specific GLMs and actual accuracy to early/late targets. Individual participants’ differences are depicted by dots, error bars represent the SEM and dashed lines represent 0 (no difference).

The stimuli and experimental procedures were the same as those in Experiment 1 (Figure 2), with the following differences. To make the task more difficult, the timings of the stimuli were shortened. The duration of the streams was 1500 ms, with the early target appearing at 500 ms and the late target appearing at 1000 ms from stream onset. All coloured circles (targets and distractors) appeared for 50 ms, and the ISIs (masks) lasted a minimum of 45 ms and a maximum of 350 ms (centred on a mean duration of 130 ms). The masks were created by sampling 16 × 16 elements from a uniform distribution of target and distractor colours and white, thus amplifying their masking effect. To ensure that the results found in Experiment 1 did not depend on specific colours, we chose four slightly different, also equally spaced colours (green: #00ED82, orange: #FFAC00, pink: #FF62FF, blue: #00DEFF) for Experiment 2.

Experiment 2 was divided into 8 blocks, each containing 72 trials and lasting ~5 min. The first block provided participants with a chance to practice the task. Participants were instructed to rest between blocks. The task lasted around 1 h in total.

At the end of the session, participants were asked to report any patterns they had noticed regarding the appearance of the stimuli but were not prompted to provide more details as in Experiment 1.

#### Data analysis

Data analysis followed the same procedures as in Experiment 1. An average of 2.05% (SD: 2.27%) trials were removed per participant for the RT analyses. An average of 0.017% of trials (range: 0.002–0.09%) constituted false alarm trials and these were not further analysed.

### Results

As in Experiment 1, our hypotheses in Experiment 2 pertained to whether participants’ performance would be improved by the temporal predictability of the targets embedded within the streams.

To address this question, we estimated values of RT and accuracy at the expected target times based on the random-target onsets and compared them to the actual RT and accuracy values for early and late targets across participants (Figure 2). A 2 × 2 ANOVA revealed a main effect of time (F(1,22) = 9.47, **p = .006, η^2^ = .037) and a main effect of predictability (F(1,22) = 6.43, *p = .02, η^2^ = .038) on RT, but no interaction between the factors (F(1,22) = 3.9, p = .06, η^2^ = .008). Additionally, we found a main effect of both time (F(1,22) = 5.64, *p = .03, η^2^ = .043) and predictability (F(1,22) = 5.56, *p = .03, η^2^ = .076) on accuracy but no interaction between the factors (F(1,22) = .02, p = .88, η^2^ < .000). From this, we concluded that participants were faster and more accurate at detecting temporally expected targets than temporally unexpected ones.

Across the trial duration, performance became faster but less accurate for long compared to short target onsets. To investigate potential changes to performance as a function of time, we calculated IES for early and late targets and found no evidence for statistically significant differences between conditions (t(22) = –0.62, p = 0.52, d = .14). Additionally, an ANOVA of random target IES with bins as factors showed that there were no differences in IES as a function of time in stream (F(1,22) = 0.43, p = .52, η^2^ = .02; **Supplementary Figure 4**). From this, we concluded that overall performance did not change systematically over time in Experiment 2.

These results were similar in participant-specific GLMs with non-linear link functions (see **Supplementary Figure 3**) and in two exhaustive GLMMs for RT (*early*: β = –.042, t = –2.19, *p = .03; *late*: β = –.013, t = –.93, p = .35) and accuracy (*early*: β = .056, z = 2.55, *p = .01; *late*: β = –.053, t = 1.92, p = .06). The small disparities observed in the results revealed by each statistical approach likely arise from differences in the treatment of participant-specific foreperiod effects in each kind of model. In the post-task debriefs of Experiment 2, no participants reported being aware of the regularities.

In Experiment 2, we replicated the main pattern of findings from Experiment 1. Namely, visual temporal expectation facilitated performance despite spatial uncertainty and when participants were unable to prepare one action. Therefore, the effects generalised across online vs. in-person testing conditions, stimulus timings, stimulus colours, and task difficulty. Furthermore, increasing the demands of stimulus presentation parameters in Experiment 2 revealed that the effects of visual temporal expectation occurred both in participants’ reaction times and accuracies.

## Discussion

In the present study, we show that expectations arising from incidentally learned temporal regularities can guide behaviour in a flexible, goal-driven manner, even in the absence of spatial and motor certainty. We replicated this finding across two experiments that differed in stimulus timings and colours, difficulty, and testing modality (online vs. in-person). Across both experiments, we observed behavioural effects of temporal expectation on participants’ reaction times. Following an increase in task difficulty in Experiment 2, the behavioural benefit was also evident in accuracy measures. Performance benefits from temporal expectations in both experiments surpassed and did not interact with behavioural effects related to the passage of time in a trial (foreperiod).

It has previously been proposed that attention based on temporal expectation must act through other stimulus attributes, particularly through space and/or motor programs (e.g., Salet et al., 2021; Thomaschke & Dreisbach, 2013). Most studies to date have investigated temporal attention under spatial or motor certainty (but see van Ede et al., 2020; Xu et al., 2021; Pratt & Abrams, 1999). Investigating the co-dependence of spatial and temporal expectation specifically, Rohenkohl et al., 2014 simultaneously cued a location (left vs. right) and a temporal interval (short vs. long) in each trial and found that behavioural benefits of visual temporal expectation were restricted to the attended location.

In contrast, in our task, no spatial certainty was required to express the benefits of temporal expectation. Here, temporal predictability was linked to stimulus colour while remaining decoupled from stimulus location and motor response. The spatially distributed nature of the benefits conferred by combined temporal-colour predictions suggests that temporal regularities can also tune feature-based attention (Cohen & Maunsell, 2011; Gabay & Henik, 2010; Kingstone, 1992; Maunsell & Treue, 2006; Pratt & Abrams, 1999; Thomaschke et al., 2016; Wagener & Hoffmann, 2010; Warren et al., 2014). We speculate that temporal expectations can latch onto different types of top-down attentional signals, including spatially distributed feature-based representations and not necessarily requiring full spatial or motor certainty.

From a neural perspective, temporal expectations have been suggested to exert their effects by amplifying neural motor and/or perceptual representations at specific times (Nobre & Rohenkohl et al., 2014; Nobre & van Ede, 2018). Preparatory motor responses have repeatedly been shown to change with temporal expectancy (Boettcher et al., 2021; Calderon et al., 2018; Cisek, 2007; Heideman, et al., 2018, 2018a, 2018b; Miniussi et al., 1999; Thomaschke & Dreisbach, 2013; Trillenberg et al., 2000; van Ede et al., 2020; van Elswijk et al., 2007; Wang et al., 2020). In the visual domain, temporal expectation has been shown to modulate neuronal firing based on sensory and sensorimotor spatial receptive fields (Ghose & Maunsell, 2002; Janssen & Shadlen, 2005), as well as other visual features (Warren et al., 2014; Anderson & Sheinberg, 2008). Our results suggest that temporal expectation can equally modulate top-down attentional biases of non-spatial features such as colour. A related observation was that by Lima et al., 2011. In their study, non-human primates (NHPs) temporally anticipated a centrally presented grating. Neuronal modulation of orientation-specific activity by temporal expectation extended beyond the spatial confines of the fovea representation, possibly reflecting how temporal predictions interact with a spatially distributed representation of orientation. In addition to modulating neural representations over at least two locations, the results of the present study speculatively suggest that temporal expectations can increase temporal preparedness for two different actions simultaneously (see also Calderon et al., 2018; Cisek, 2007; Kornysheva et al., 2013; Wang et al., 2020).

In our task, target locations and corresponding responses were simple and not in strong competition; the two locations were in different hemifields, and the two responses engaged different hands. It will be important to investigate whether benefits from temporal orienting of attention still occur under greater spatial and motor ambiguity or competition. It remains possible that temporal expectation is confined to influencing non-competing spatial or motor representations that can be activated concurrently to guide task performance. In addition, spatial or motor factors may interact strongly with other non-spatial factors in setting proactive expectations and set limits to how learned temporal structures can influence the resulting top-down attentional signals.

An innovative aspect of the present design is the orthogonal manipulation of task relevance (task-relevant and task-irrelevant targets) and temporal expectations (early, late, and random; see also Duyar et al., 2023). Most informative cueing studies of temporal attention manipulate expectations by changing the probability of target appearance (e.g., Coull & Nobre, 1998; Miniussi et al., 1999). In contrast, here, we manipulate task goals on a trial-by-trial basis, such that all or most targets are present in any given trial, but only one is relevant to the task at hand (see also Denison et al., 2017, 2021; Fernández et al., 2019; Griffin et al., 2002).

In real-world contexts, goals and contextual regularities work together to guide attention proactively. Yet, experimentally, deployment of attention based on goals (e.g., Griffin et al., 2002) or contextual regularities (e.g., Olson & Chun, 2001) have been investigated separately using distinct task designs. Our mixed design brings the two types of guidance signals together: expectations result from incidentally learned temporal structures and goals determine their flexible utilization.

While our study suggests that a combination of contextual temporal expectations and task goals can be used to guide behaviour, it does not elucidate how they operate together to guide attention. For example, the present studies are uninformative regarding the extent to which temporal expectations modulate processing of predictable but task-irrelevant targets. In the future, this task design could be combined with continuous and time resolved metrics such as gaze and pupil size (see Denison et al., 2019, 2020; Palmieri et al., 2023), neurophysiological measures of anticipation (e.g., CNV, alpha power) and stimulus processing (e.g., P1, N2PC, P300, decoding of target colour), to probe how temporal regularities enhance and/or inhibit the processing of goal-relevant and goal-irrelevant events over time.

Furthermore, the mechanisms whereby temporal regularities are learned and used to guide visual attention in the present task require further study. Participants likely formed associations between specific colours and moments in time, which in turn facilitated target identification and localization to select the appropriate response (see also Chun & Jiang, 1998; Zhao et al., 2013). From a neural perspective, the incidental learning of temporal structure through statistical regularities may include visual areas interacting with the basal ganglia, the cerebellum and/or the hippocampus-based learning systems (e.g., Breska & Ivry, 2016; Chun & Phelps, 1999; Covington et al., 2018; Goldfarb et al., 2016; Turk-Browne et al., 2009).

Other than their online vs. in-person implementation, the main difference between Experiments 1 and 2 was the increased temporal competition and resulting task difficulty of the latter. This resulted in accuracy improvements for identifying temporally expected targets. Some studies have suggested that the speeding of responses caused by temporal expectations is reflective of modulations in preparatory motor activity, whereas changes in accuracy reflect influences on perceptual processes (e.g., Tal-Perry & Yuval-Greenberg, 2022). In the context of the present task, this would speculatively point to a modulation of both motor and perceptual processes when task difficulty is sufficiently high (see also Kingstone, 1992). Nevertheless, titrating what stages of processing (sensory, associative, or motor) contribute to our behavioural effects would benefit from using additional appropriate psychophysical methods (e.g., signal detection theory, theory-of-visual-attention modelling; e.g., Vangkilde et al., 2013) and electrophysiological measures.

Investigating cognitive processes unfolding in extended contexts brings along difficulties. The passage of time (foreperiod) may drive expectation and/or change the level of non-specific motor preparation or arousal, hence resulting in systematic changes to the behavioural metrics of interest (Los, 2010; Luce, 1991). Like previous studies, we sought to reduce foreperiod-related effects by keeping the conditional temporal probability of random targets appearing fixed at 50% in each successive third. Foreperiod effects were nevertheless present in the behavioural metrics of this baseline condition. Understanding the source of the foreperiod effect under such controlled conditions is of intrinsic interest and deserved investigation. In our case, we modelled the foreperiod effect by capitalizing on temporal spread of random target appearance to isolate the benefits of temporal attention above and beyond the foreperiod effects.

The complications observed in dynamic tasks tap into the nuances of natural behaviour unfolding in real-world contexts in which several dimensions of temporal flux co-exist and interact (Nobre & van Ede, 2023). Our results highlight the need to develop dynamic, ecologically valid designs in which foreperiod effects and other kinds of temporal expectations co-occur and simultaneously influence behaviour.

Taken together, our results show that visual temporal expectations formed on the basis of incidentally learned temporal regularities can be called upon flexibly, in a goal-driven manner, to guide behaviour. Moreover, we find that temporal attention can facilitate performance in the absence of concomitant spatial or motor expectations in dynamically unfolding environments. Combining this task design with continuous behavioural and neurophysiological measures can pave the way to a better understanding of temporal attention.

## Data Accessibility Statement

Analysis scripts and data can be accessed here [https://osf.io/p2gvh/].

## Additional File

The additional file for this article can be found as follows:

10.5334/joc.360.s1 Supplementary Figures. Figures 1 to 4.
