# Supplementary material for: Goal-Dependent Use of Temporal Regularities to Orient Attention under Spatial and Action Uncertainty
Source: J Cogn. 2024 Apr 25;7(1):37. doi: 10.5334/joc.360 (PMC11049616; doi:10.5334/joc.360)
Supplement: Supplementary Figures. — Figures 1 to 4. [file joc-7-1-360-s1.pdf]

Supplementary Figures

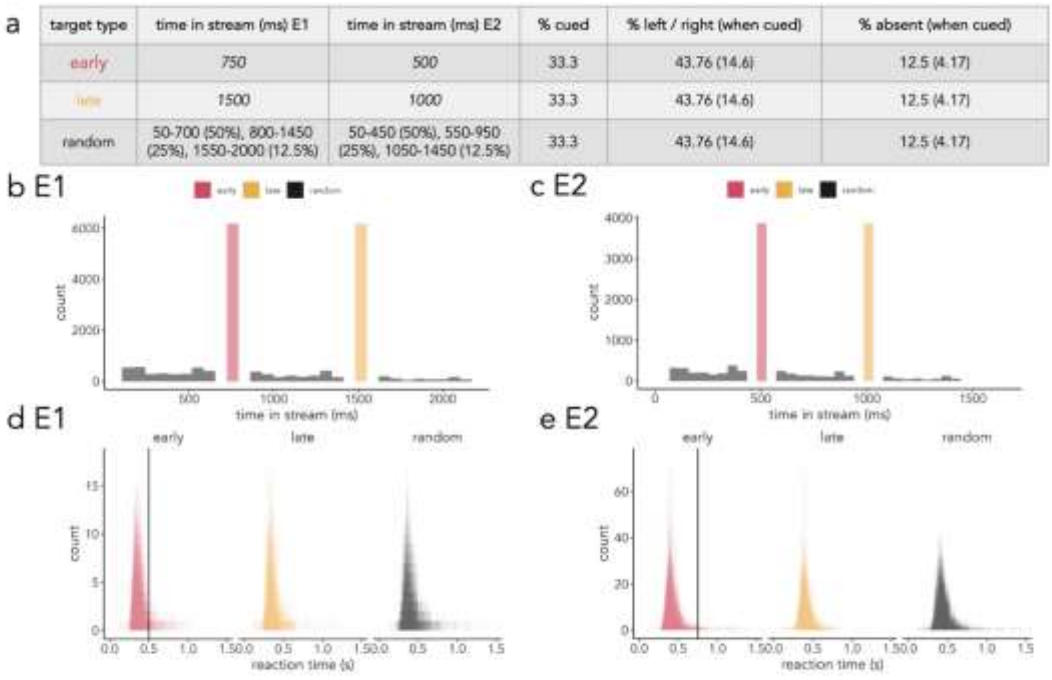

target type	time in stream (ms) E1	time in stream (ms) E2	% cued	% left / right (when cued)	% absent (when cued)
early	750	500	33.3	43.76 (14.6)	12.5 (4.17)
late	1500	1000	33.3	43.76 (14.6)	12.5 (4.17)
random	50-700 (50%), 800-1450 (25%), 1550-2000 (12.5%)	50-450 (50%), 550-950 (25%), 1050-1450 (12.5%)	33.3	43.76 (14.6)	12.5 (4.17)

Supplementary Figure 1. Details about trial types and proportions, the timing of targets, and RT distributions. a) Details about trial types and percentages. In each trial, participants were cued and asked to respond to one of three targets which differed on their timing (early, late, and random) and colour. One of three targets was cued in each trial and each target type (early, late, and random) was cued in one third of trials. Thus, each target type was task-relevant only in 33.4% of trials. Each target was absent in 12.5% of all trials and only one target was absent in each trial, thus all three targets were present in 62.5% of trials. Out of the 12.5% of trials in which each target was absent, only a third required a response to the absent target and, consequently, was a no-response trial. Thus, only 12.5% of trials were no-response trials, a third per target type (early, late, and random). Each target type (early, late, and random) appeared on either side equiprobably. b) Count of trials in which early, late, and random targets appeared at specific times from stream onset (0 s) in Experiment 1. c) Count of trials in which early, late, and random targets appeared at specific times from stream onset (0 s) in Experiment 2. d) Histogram of reaction times (in s) to early, late, and random targets across participants in Experiment 1. The vertical line on the first panel reflects the time of late target appearance. e) Histogram of reaction times (in s) to early, late, and random targets across participants in Experiment 2. The vertical line on the first panel reflects the time of late target appearance.

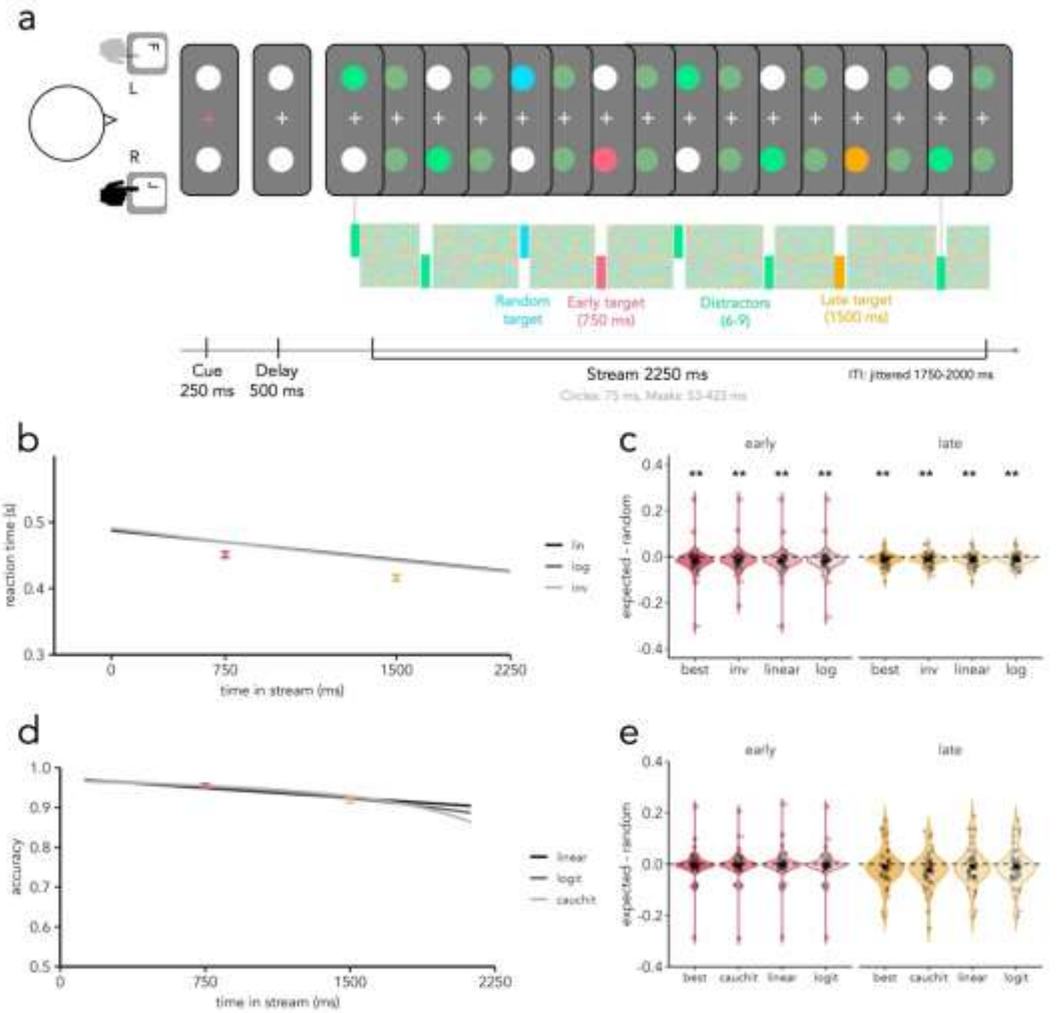

Supplementary Figure 2. Online task design, accuracy, and RT results using GLMs with different link functions. a) Design of the online experiment. At the beginning of each trial, participants saw a cue (change in the colour of the fixation cross) indicating which out of three coloured targets to detect. Participants had to search for a circle with this colour within a stream of bilaterally appearing circles and masks. Unknown to the participants, one of these targets appeared at 750 ms from stream onset (early target), another appeared at 1500 ms from stream onset (late target) and the other could happen at any time (random target). Participants had to respond with the hand corresponding to the side of target appearance. b) GLM fits of RT to random targets as a function of target onset and mean RT to early (pink) and late (yellow) targets across participants (error bars represent standard error of the mean). Differently coloured lines represent the GLM fits with the different link functions: linear, logarithmic, and inverse. c) Difference between RT at the time of early/late target as estimated from participant-specific GLMs (linear, logarithmic, inverse, and participant-specific best fit) and actual RT to early/late targets. Results from 2x2 ANOVAs with time and predictability as factors for the different fits. Best fit: main effect of time ($F(1,48) = 27.77$, ***$p < .001$, $\eta^2 = .04$), a main effect of predictability ($F(1,48) = 8.14$, **$p = .006$, $\eta^2 = .01$) and no interaction between the factors ($F(1,48) = .1$, $p = .7$, $\eta^2 < .000$). Inverse fit: main effect of time ($F(1,48) = 31$, ***$p < .001$, $\eta^2 = .044$), amain effect of predictability ($F(1,48) = 7.92$, **$p = .007$, $\eta^2 = .008$) and no interaction between the factors ($F(1,48) = .05$, $p = .8$, $\eta^2 < .000$). Logarithmic fit: main effect of time ($F(1,48) = 27.66$, ***$p < .001$, $\eta^2 = .044$), a main effect of predictability ($F(1,48) = 8.84$, **$p = .005$, $\eta^2 = .01$) and no interaction between the factors ($F(1,48) = .1$, $p = .7$, $\eta^2 < .000$). d) GLM fits of accuracy to random targets as a function of target onset and mean accuracy to early (pink) and late (yellow) targets across participants (error bars represent standard error of the mean).

Differently coloured thick lines represent GLM fits with the different link functions: linear, logit, and cauchit. e) Difference between accuracy at the time of early/late target as estimated from participant specific GLMs (linear, logit, cauchit, and participant-specific best fit) and actual accuracy to early/late targets. Results from 2x2 ANOVAs with time and predictability as factors for the different fits. Best fit: main effect of time ($F(1,48) = 15.1$, ***$p < .001$, $\eta^2 = .06$), no main effect of predictability ($F(1,48) = 0.8$, $p = .38$, $\eta^2 = .003$) and no interaction between the factors ($F(1,48) = .027$, $p = .6$, $\eta^2 < .000$). Cauchit fit: main effect of time ($F(1,48) = 14.27$, ***$p < .001$, $\eta^2 = .045$), no main effect of predictability ($F(1,48) = 3.16$, $p = .08$, $\eta^2 = .01$) and an interaction between both factors ($F(1,48) = 4.34$, *$p = .04$, $\eta^2 = .005$). Logit fit: main effect of time ($F(1,48) = 15.1$, ***$p < .001$, $\eta^2 = .06$), no main effect of predictability ($F(1,48) = 0.78$, $p = .37$, $\eta^2 = .003$) and no interaction between the factors ($F(1,48) = .027$, $p = .6$, $\eta^2 < .000$).

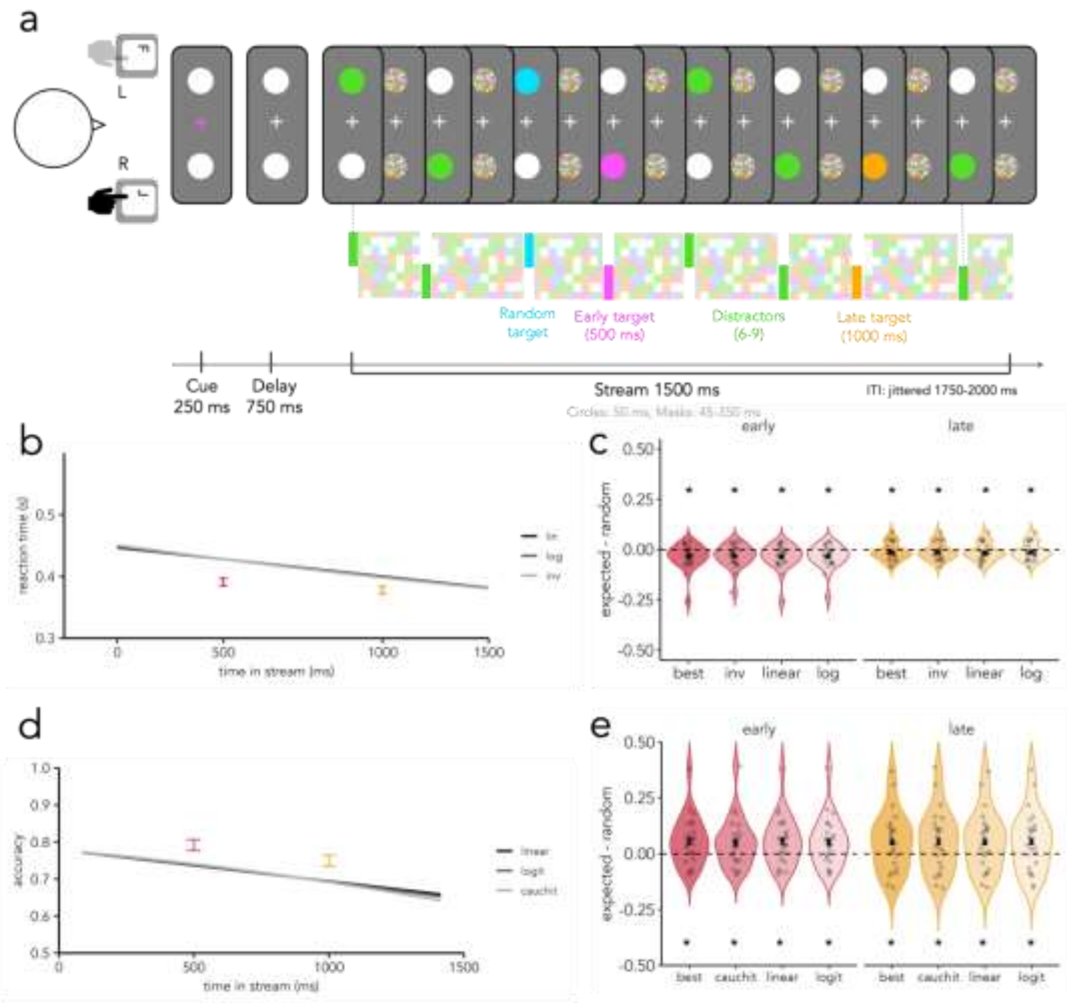

Supplementary Figure 3. In-person task design, accuracy, and RT results using GLMs with different link functions. a) Design of the in-person experiment. At the beginning of each trial, participants saw a cue (change in the colour of the fixation cross) indicating which out of three coloured targets to detect. Participants had to search for a circle with this colour within a stream of bilaterally appearing circles and masks. Unknown to the participants, one of these targets appeared at 500 ms from stream onset (early target), another appeared at 1000 ms from stream onset (late target) and the other could happen at any time (random target). Participants had to respond with the hand corresponding to the side of target appearance. b) GLM fits of RT to random targets as a function of target onset and mean RT to early (pink) and late (yellow) targets across participants (error bars represent standard error of the mean). Differently coloured lines represent GLM fits with the different link functions: linear, logarithmic, and inverse. c) Difference between RT at the time of early/late target as estimated from participant specific GLMs (linear, logarithmic, inverse, and participant-specific best fit) and actual RT to early/late targets. Results from 2x2 ANOVAs with time and predictability as factors for the different fits. Best fit: main effect of time ($F(1,22) = 9.7$, $^{**}p = .005$, $\eta^2 = .037$), main effect of predictability ($F(1,22) = 5.83$, $^{*}p = .02$, $\eta^2 = .034$) and no interaction between both factors ($F(1,22) = 4.19$, $p = .05$, $\eta^2 = .008$). Inverse fit: main effect of time ($F(1,22) = 9.8$, $^{**}p = .004$, $\eta^2 = .038$), a main effect of predictability ($F(1,22) = 5.7$, $^{*}p = .02$, $\eta^2 = .03$) and an interaction between both factors ($F(1,22) = 4.5$, $^{*}p = .045$, $\eta^2 = .008$). Logarithmic fit: main effect of time ($F(1,22) = 9.66$, $^{**}p = .005$, $\eta^2 = .038$), main effect of predictability ($F(1,22) = 6.11$, $^{*}p = .02$, $\eta^2 = .033$) and no interaction between the factors ($F(1,22) = 4.28$, $p = .05$, $\eta^2 = .008$). d) GLM fits of

accuracy to random targets as a function of target onset and mean accuracy to early (pink) and late (yellow) targets across participants (error bars represent standard error of the mean). Differently coloured lines represent GLM fits with the different link functions: linear, logit, and cauchit. e) Difference between accuracy at the time of early/late target as estimated from participant specific GLMs (linear, logit, cauchit, and participant-specific best fit) and actual accuracy to early/late targets. Results from 2x2 ANOVAs with time and predictability as factors for the different fits. Best fit: main effect of time ($F(1,22) = 5.67$, $*p = .02$, $\eta^2 = .043$), a main effect of predictability ($F(1,22) = 5.52$, $*p = .03$, $\eta^2 = .07$) and no interaction between the factors ($F(1,48) = .02$, $p = .89$, $\eta^2 < .000$).. Cauchit fit: main effect of time ($F(1,22) = 6.55$, $*p = .02$, $\eta^2 = .05$), a main effect of predictability ($F(1,22) = 4.88$, $*p = .04$, $\eta^2 = .067$) and no interaction between the factors ($F(1,48) = .035$, $p = .85$, $\eta^2 < .000$). Logit fit: main effect of time ($F(1,22) = 6.1$, $*p = .02$, $\eta^2 = .046$), a main effect of predictability ($F(1,22) = 5.35$, $*p = .03$, $\eta^2 = .074$) and no interaction between the factors ($F(1,48) = .0003$, $p = .98$, $\eta^2 < .000$).

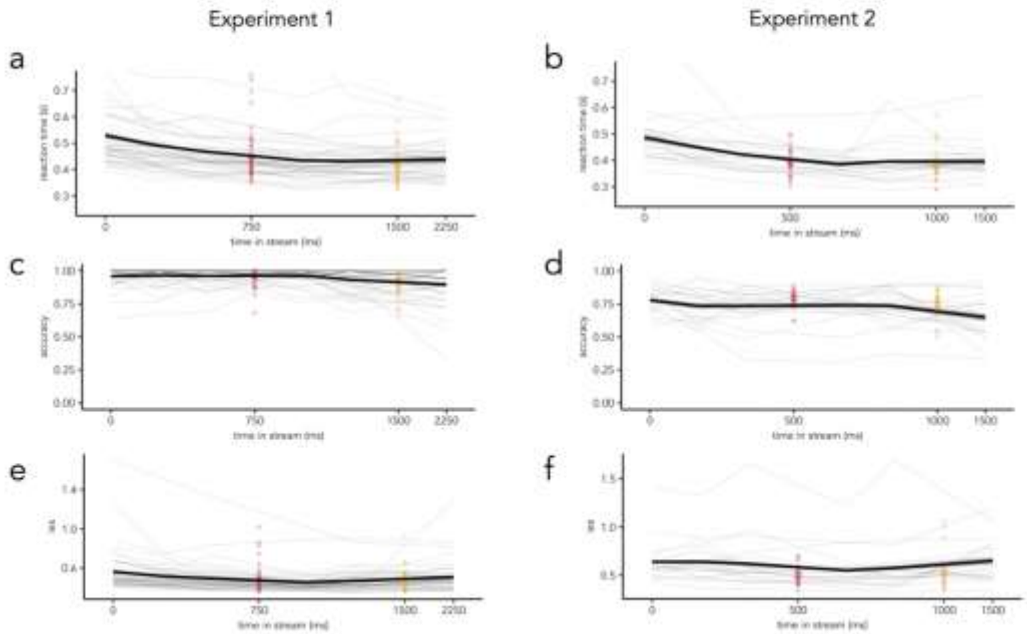

Supplementary Figure 4. Reaction time (s), accuracy and inverse efficiency score (IES) as a function of binned time-in-stream in Experiments 1 (left column) and 2 (right column). Time-in-stream was split into 8 bins corresponding to targets that occurred in the following bins: *Experiment 1*: 0-0.24 s, 0.24-0.44 s, 0.44-0.7 s, 0.7-0.8 s (early), 0.8-1.1 s, 1.1-1.45 s, 1.45-1.55 s (late) and 1.55-2.25. *Experiment 2*: 0-0.15 s, 0.15-0.3 s, 0.3-0.45 s, 0.45-0.55 s (early), 0.55-0.75 s, 0.75-0.95 s, 0.95-1.05 s (late) and 1.05-1.5 s. RT (a, b), accuracy (c, d), and a metric which combines both, the inverse efficiency score (IES; c, d; Townsend & Ashby, 1983) was calculated in each bin and plotted for each participant in the random (gray lines), early (pink dots) and late (yellow dots) conditions. The participant-average for the random (thick black line), early (pink) and late (yellow) conditions is shown with error bars and shaded areas representing the standard error of the mean. A paired sample t-test of *IES* values for early and late targets across participants revealed no significant differences between the temporally expected targets (*Experiment 1*: t(48) = 1.47, p = 0.15, d = .21; *Experiment 2*: t(22) = -0.62, p = 0.52, d = .14). An ANOVA of IES for the random target with bins as factors revealed a slight tendency for an effect of bins on IES in Experiment 1 (F(1,48) = 4.36, *p = .04, η^2 = .083) which did not survive multiple comparison correction for this unplanned contrast and in Experiment 1 and no significant effect of bin in IES for random targets in Experiment 2 (F(1,22) = 0.43, p = .52, η^2 = .02).
